# Primary leiomyosarcoma of the scalp: a case report and review of the literature

**DOI:** 10.3389/fonc.2025.1533114

**Published:** 2025-02-11

**Authors:** Shuo Gao, Pule Liu, Jixing Liu, Wenzhen Yang, Shude Yang

**Affiliations:** Department of Neurosurgery, The Second Hospital & Clinical Medical School, Lanzhou University, Lanzhou, Gansu, China

**Keywords:** leiomyosarcoma, sarcoma, scalp, surgery, case report

## Abstract

**Background and importance:**

Leiomyosarcoma is a rare and aggressive malignant tumor with a high potential for relapse and metastasis. Correct and timely diagnosis is critical for effective treatment, yet it is often challenging due to the diverse clinical presentations. This case report highlights the significance of early identification and the consequences of delayed diagnosis in scalp leiomyosarcoma.

**Clinical presentation:**

We present the case of a 39-year-old woman with a scalp neoplasm. Initially, the diagnosis was missed, leading to a delay in surgical intervention. The tumor demonstrated a locally aggressive course, infiltrating the skull and dura mater. Upon admission, the scalp tumor was promptly excised. This case provides valuable insights into the varied symptoms and presentations of scalp leiomyosarcoma, which can aid in the recognition of this condition.

**Conclusion:**

This report underscores the importance of considering leiomyosarcoma in the differential diagnosis of scalp masses, particularly when the etiology is unclear. Early recognition and intervention are essential to prevent locally invasive growth and potential metastasis, emphasizing the need for a high index of suspicion among healthcare professionals.

## Introduction

Leiomyosarcoma (LMS) is a malignant tumor that originates from smooth muscle cells and constitutes a relatively rare subtype of soft tissue sarcomas (STS) (1, 2). According to the currently available epidemiological data, head and neck soft tissue sarcomas (HNSTS) account for only 5%-10% of all STS. Within this category, head and neck leiomyosarcoma constitutes approximately 7% of HNSTS (3). This disease is more prevalent among middle-aged individuals; however, its diverse clinical presentations and low incidence rate often result in clinicians having limited understanding, potentially leading to misdiagnosis or delayed diagnosis. This case report and literature review aims to examine the epidemiological, clinical, anatomical, treatment, and prognostic aspects of LMS.

## Case report

**Figure 1:**
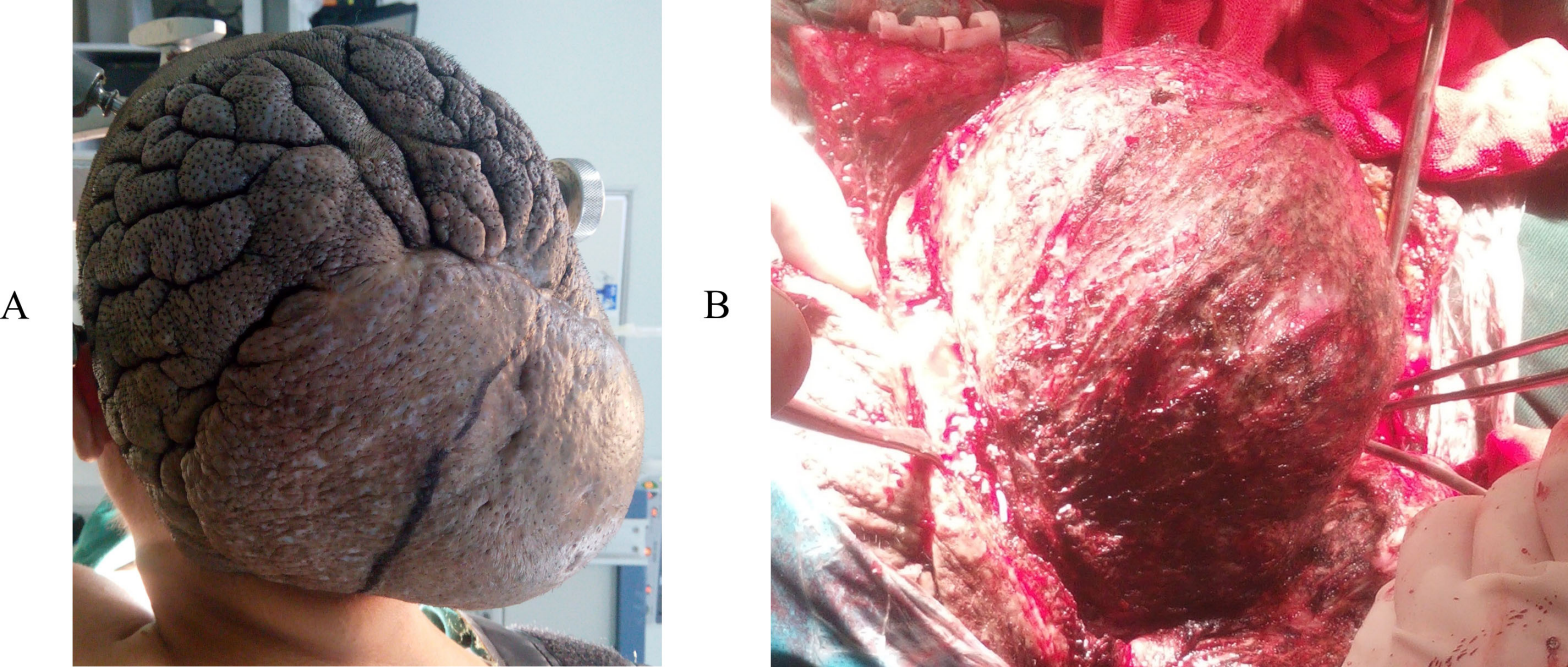
**(A)** Local cutis verticis gyrata and a 12cm hard, painless mass in the occipital and vertex region. The lesion was burgeoning. The tumor was exposed during the operation **(B)**.

**Figure 2:**
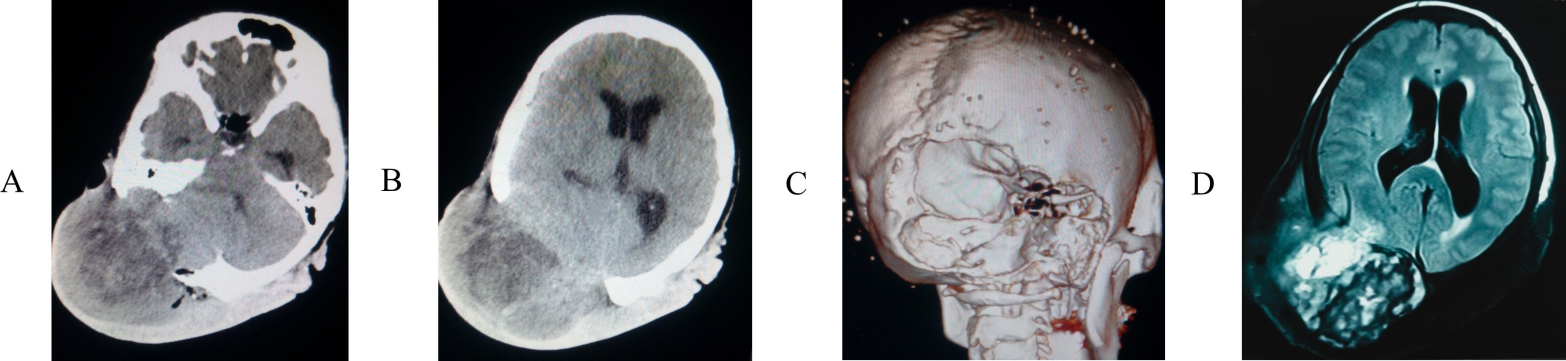
The computed tomography (CT) scan **(A, B)** showed that the tumor located on the right side of the occipital and vertex region, 3D reconstruction **(C)** revealed the skull was severely destroyed. **(D)** Magnetic resonance imaging (MRI) revealed the tumor signal was mixed and that neighboring brain tissue was compressed, poorly circumscribed lesion with infiltrative border.

**Figure 3:**
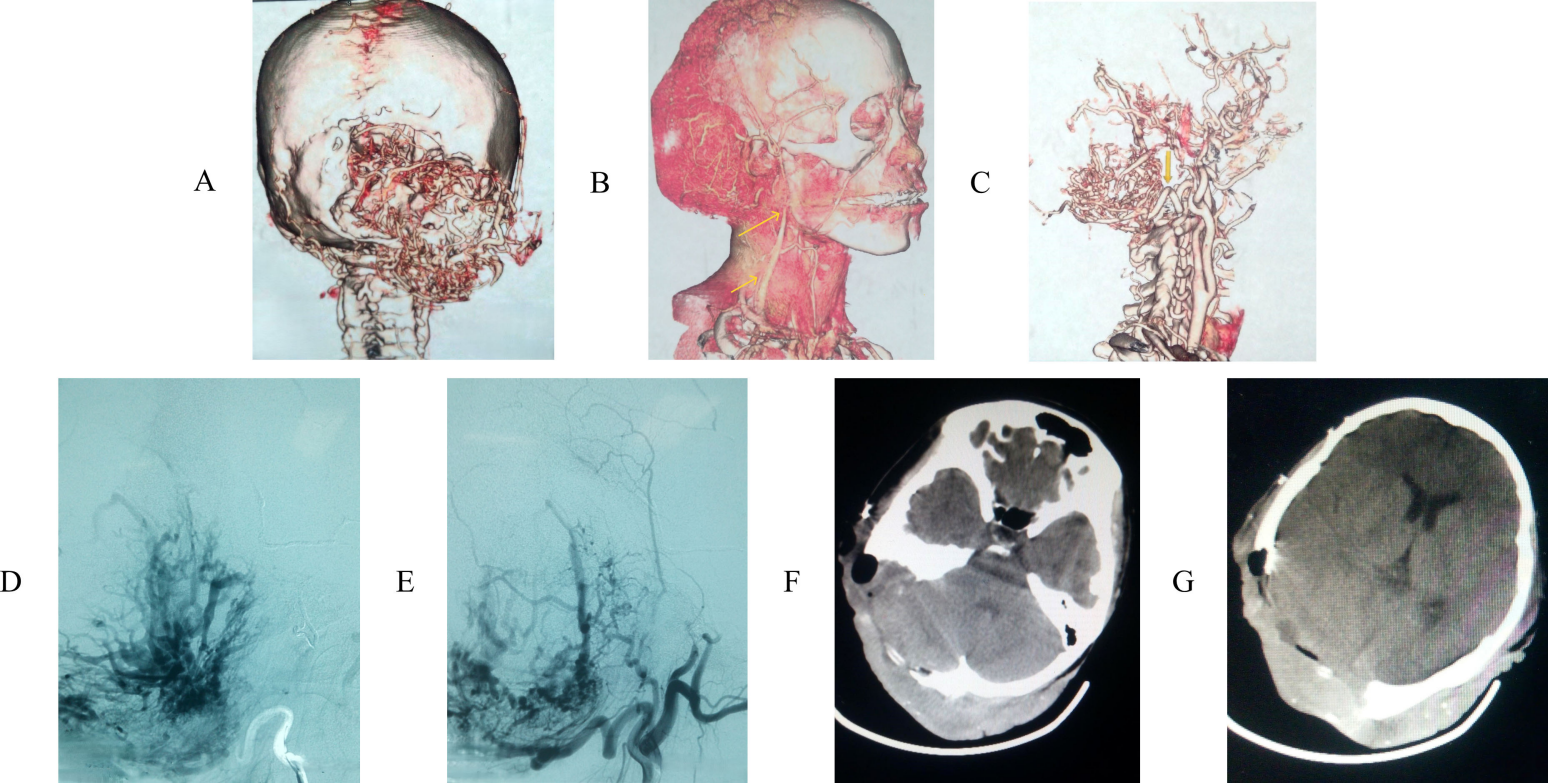
**(A)** CT angiography (CTA) with 3D reconstruction revealed tumor was hypervascula, leading to an angiomatous lesion with skull invasion. **(B)** CTA presented the lesion’s vascularization. **(C)** the lesion’s main vascularization were occipital artery (bottom arrow) and arteriae auricularis posterior (upper arrow). **(D, E)** the patient accepted embolization therapy of occipital. **(F, G)** most parts of the tumor were removed.

A 39-year-old woman presented to our hospital for the first time with a scalp mass accompanied by headaches and dizziness. The patient reported that 15 years ago, she first discovered a painless, soft mass approximately 3 centimeters in diameter on the occipital scalp. During this period, the patient underwent a Computerized tomography (CT) scan at a local hospital, which revealed a diagnosis of scalp hemangioma. Consequently, due to the patient’s impoverished family circumstances, surgical treatment was not financially viable at that time, and no additional treatments were pursued. Over the past year, the diameter of the lesion rapidly increased to 12 centimeters and gradually became hard. In addition, the patient has no family history and psychosocial background, as well as any history of associated genetic conditions. Concurrently, she experienced intermittent, stabbing headaches primarily in the occipital region, as well as vertigo characterized by a spinning sensation and unsteadiness while standing. Physical examination revealed localized scalp folds and a hard, painless mass measuring 12 centimeters in diameter on the occipital and parietal regions (**Figure 1**). CT with 3D reconstruction showed that the tumor was located in the right occipital and parietal areas, associated with severe bone destruction (**Figures 2A-C**). The lesion extended from the scalp to the dura mater. Although magnetic resonance imaging (MRI) revealed mixed tumor signals and compression of the adjacent brain (**Figure 2D**), it remained uncertain whether the brain parenchyma was invaded. CT angiography (CTA) with 3D reconstruction demonstrated the vascularity of the lesion, indicating that the tumor was hypervascular (**Figures 3A-C**). The CTA findings suggested that the lesion might be an hemangioma. To obstruct the tumor’s blood supply, the patient underwent occipital artery embolization (**Figures 3D, E**). Seventy-two hours post-embolization, the majority of the tumor was excised (**Figures 3F, G**); although the dura mater was invaded, several portions near the transverse sinus or sigmoid sinus remained intact, and the tumor did not invade the brain parenchyma. In addition to hemangioma, we also considered the following differential diagnoses: neurogenic tumors (such as neurofibroma and schwannoma), trichilemmoma, basal cell carcinoma and squamous cell carcinoma, lipoma and liposarcoma, fibroma and fibrosarcoma, malignant fibrous histiocytoma, rhabdomyosarcoma, and metastatic disease, particularly from melanoma or other common cancers. However, based on the lesion’s location, the patient’s clinical presentation, radiological features (including the tumor’s morphology, margins, density/signal characteristics, and enhancement pattern), as well as the patient’s medical history and risk factors, our preliminary clinical impression suggests that the tumor may be an atypical malignant neoplasm originating from a hemangioma.

**Figure 4:**
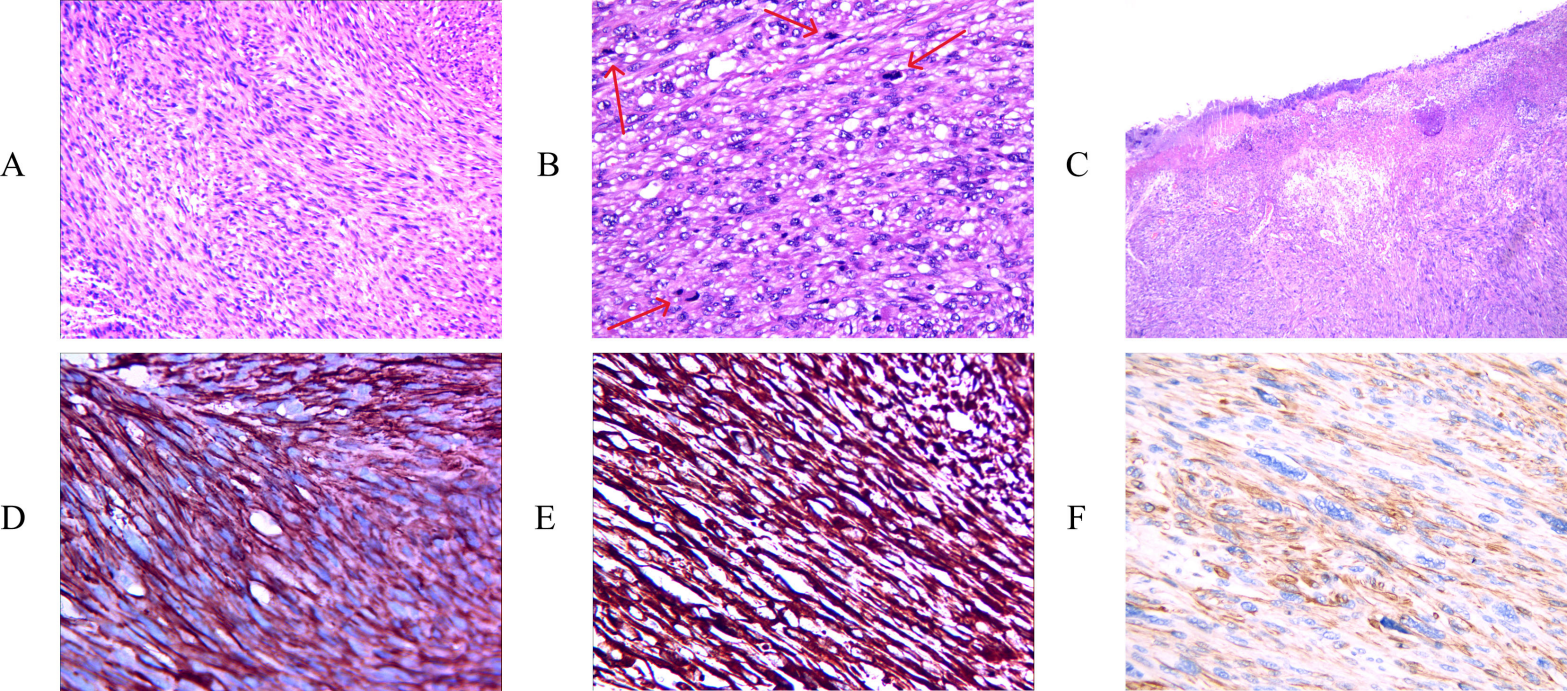
H&E staining showing interlacing fascicles of elongated spindle cells with cytological atypia and abundant eosinophilic cytoplasm [**(A)** X100]. High-power microscopic examination showing moderate mitosis. The arrows are annotated as mitosis. [**(B)** X200], marked nuclear atypia and tumoral necrosis [**(C)** X40]. Immunohistochemical staining of, SMA [**(D)** X400], and vimentin [**(E)** X400], H-caldesmon [**(F)** X400] were positive.

The histopathological results (**Figure 4A**) revealed that the tumor consisted of interwoven bundles of spindle cells exhibiting marked cytological atypia and eosinophilic cytoplasm. Key findings included densely stained nuclei, moderate mitotic activity (with a mitotic count of 10 per 10 high-power fields (HPFs)), and less than 50% of the observed area showing tumor necrosis (**Figures 4B, C**). The expression level of Ki67 was determined to exceed 15%. Immunohistochemical staining revealed diffuse expression of H-calmodulin, smooth muscle actin (SMA), and vimentin in the tumor cells, with a positive result for H-calmodulin (**Figures 4D–F**). Expression of estrogen receptor (ER), progesterone receptor (PR), epithelial membrane antigen (EMA), CD34, desmin, SMA, Bcl-2, and myoglobin were all negative. The differential diagnoses in histopathology primarily encompassed other spindle cell lesions, including spindle cell carcinoma, desmoplastic melanoma, superficial fibular sarcoma, malignant peripheral nerve sheath tumor, and vascular tumors. For the diagnosis of leiomyosarcoma (LMS), at least two muscle-specific immunohistochemical markers are necessary for confirmation, and in our examination, three markers exhibited diffuse expression. Furthermore, to rule out other spindle cell lesions, immunohistochemical staining was conducted, which included markers such as ER, PR, EMA, CD34, desmin, SMA, Bcl-2, and myoglobin, all of which were negative in leiomyosarcoma (LMS). Consequently, in light of the pathological findings and the patient’s prolonged history of a scalp mass, the tumor was classified as a primary scalp leiomyosarcoma.

Because it was a malignant sarcoma, the patient received radiation therapy and chemotherapy in the hospital one month after the operation. Due to the lack of a comprehensive systemic examination, the presence of tumor metastasis remains uncertain. However, over the past four months, the patient has not reported any symptoms related to tumor invasion or metastasis. Furthermore, significant abnormalities were not found on CT scans of the head and chest.

## Discussion

Leiomyosarcoma (LMS) is an extremely rare malignant tumor of smooth muscle origin, with an incidence below two per million (4, 5). The most common site of its occurrence is the retroperitoneal region. Superficial LMS is divided into two subtypes: cutaneous and subcutaneous (6, 7). The subcutaneous type, which grows relatively fast and usually does not result in epidermal changes such as ulceration or discoloration (8), arises from the smooth muscle lining of arterioles and veins in the subcutaneous tissue (9). Due to the scalp’s rich vascularity (10), LMS in the scalp is more likely to cause metastasis and local recurrence (3, 11). According to literature reports, the common sites of metastatic spread include the lungs, colon, kidneys, ovaries, uterine cervix, and oral cavity (12–14). This case belongs to the subcutaneous type. Physically, the lesion did not protrude beyond the dermis, and no ulceration was observed, except for local cutis verticis gyrata. Based on imaging and clinical symptoms, we have not yet found direct evidence of tumor metastasis in this patient.

Under normal conditions, LMS may present as a slowly enlarging, firm, nonulcerated, painless mass (15). Its physical appearance can be deceptive and may be mistaken for a benign tumor. Presenting signs and symptoms are nonspecific and usually correspond to the location where the tumor arises. Some scholars argue that pain is the most common symptom of LMS, occurring in 80%–95% of patients (16). Pruritus, burning, and bleeding are also common. In our case, the patient experienced a headache, dizziness, instability while standing, and no tenderness. These positive symptoms may be associated with compression of the brain by the lesion and invasion of the scalp lesion into the dura mater. Larger lesions may exhibit focal areas of hemorrhage and necrosis. According to a large review of LMS of the superficial soft tissues, lesions of soft tissue origin measuring 2.5 cm or larger are more likely to be malignant (17).

The diagnosis of this rare malignant tumor must be based on histologic and ultrastructural examination. LMS is characterized by poorly circumscribed interlacing fascicles of elongated spindle-shaped cells with prominent blunt-ended nuclei and abundant eosinophilic cytoplasm in the subcutaneous tissue (18). Immunohistochemical identification of desmin, vimentin, actin, and myoglobin is helpful in diagnosis. LMS is characterized by the co-expression of vimentin, desmin, and muscle-specific actin. Tumors that exhibit one mitotic figure per five HPFs are considered malignant (19). The diagnostic value of progesterone receptor (PR) expression is still being actively researched. PR expression is useful in distinguishing LMS from smooth muscle tumors of uncertain malignant potential (STUMP), leiomyoma (LM), and atypical leiomyoma (ALM) (20–22). Thus, PR expression may aid in effectively distinguishing both ALMs and STUMP from LMS. Desmin staining results are variable, with an inverse relationship to the tumor’s vascularity. In this case, immunohistochemical staining of H-caldesmon, smooth muscle actin (SMA), and vimentin were positive, while desmin, myoglobin, and PR expression were negative (15). Combining hematoxylin-eosin and immunohistochemical staining, this case is most consistent with LMS.

The prognosis of scalp LMS is generally good, with the five-year survival rate of noncutaneous LMS reported to be 70.5% (23). Local recurrence rates range from 20%–35%, and the prognosis may be associated with tumor site, size, grade, and mitotic figures (24). There is no uniform standard for the treatment of scalp LMS, but maximal tumor resection is the treatment of choice, and surgical treatment can significantly improve the prognosis (25). Metastatic or recurrent LMS is managed with surgery and adjuvant radiochemotherapy, though no standard treatment protocol has been established so far. But the role of adjuvant chemotherapy has not been well understood, and no overall survival advantage has been shown in prospective studies (26). The treatment approach for leiomyosarcoma typically depends on the tumor’s size, location, metastatic status, and the patient’s overall health (25). Surgical resection is the most frequently employed treatment modality. In the case of localized leiomyosarcoma, surgical intervention is the treatment of choice, with the objective of achieving complete excision of the tumor along with a margin of normal tissue to ensure a negative surgical margin. However, for superficial leiomyosarcoma, Mohs Micrographic Surgery (MMS) is more effective, with reports indicating that the recurrence rate following MMS is significantly lower than that following wide local excision (27). Radiotherapy is another modality, categorized into preoperative and postoperative applications. Preoperative radiotherapy is primarily utilized to reduce tumor size, facilitating surgical excision; postoperative radiotherapy aims to eradicate residual tumor cells that may remain after surgery, thereby mitigating the risk of local recurrence (28, 29). LMS, particularly uterine LMS, demonstrates high responsiveness to chemotherapeutic agents. Chemotherapy, typically employing doxorubicin- or gemcitabine-based regimens, is effective in controlling metastatic leiomyosarcoma and extending survival (30). Additionally, novel treatment modalities for LMS are emerging, including targeted DNA repair therapies, immunotherapies, and innovative combinations of chemical agents.

## Conclusion

Scalp LMS is an uncommon and aggressive soft tissue neoplasm that presents significant diagnostic and therapeutic challenges owing to its unique anatomical location and high rate of recurrence. Through the analysis of this clinical case of primary scalp LMS complemented by a literature review, the objective is to enhance clinicians’ comprehension of the disease and to prevent diagnostic omissions in clinical practice. Further research is warranted to improve the early diagnosis and treatment strategies for scalp LMS.

## Data Availability

The original contributions presented in the study are included in the article/supplementary material. Further inquiries can be directed to the corresponding author.
